# The Impact of Enhancing Phone Activeness on the Negative Effect Induced by the Presence of a Cell Phone

**DOI:** 10.3389/fpsyg.2022.920878

**Published:** 2022-07-06

**Authors:** Wenjuan Liu, Akihiko Dempo, Kazumitsu Shinohara

**Affiliations:** Graduate School of Human Sciences, Osaka University, Osaka, Japan

**Keywords:** the presence of a cell phone, phone activeness, attention, dual-task paradigm, 0-back, luminance detection

## Abstract

In the information-driven workplace, cell phones have gradually become irreplaceable. Although the use of work-related cell phones can bring convenience, recent research has demonstrated that the presence of a cell phone can impair cognitive task performance by reducing available attentional resources and suggested that the effect of the phone’s presence can be influenced by phone-related factors. This study focused on the relationship between this effect and phone activeness and conducted two experiments to investigate whether increasing phone activeness is associated with a stronger effect from the phone’s presence by using a dual-task paradigm (primary: letter recognition task, secondary: luminance-change detection task). Phone activeness was manipulated by two potential factors: the phone’s power state (control, powered-off, powered-on) and physical contact state (the phone was placed on the desk or held in the hand). The results showed that secondary task performance decreased with the phone’s presence, regardless of its power state and contact state. This indicated that the presence of the phone only affects the available attentional resources devoted to the peripheral visual field where the secondary task stimuli occurred; however, the effect of the phone’s presence was not moderated by phone activeness. The current findings provided several extended understandings related to the negative effects caused by the presence of the cell phone and their underlying mechanisms.

## Introduction

Recently, the cell phone has appeared in the workplace as a common information and communication device. However, a growing body of evidence has shown that the presence of the cell phone can negatively affect cognitive abilities (Thornton et al., 2014; Ito and Kawahara, 2017; Ward et al., 2017; Canale et al., 2019; Tanil and Yong, 2020), and therefore, directly undermine the outcome of cognitive activities (Przybylski and Weinstein, 2013; Misra, 2016; Crowley et al., 2018). Such studies have demonstrated that the presence of the cell phone can trigger a cognitive cost by constantly attracting attention away from the focal task and suggested that the underlying cause for this detrimental effect may be induced both externally (e.g., attention captured by calls or notifications) and internally (e.g., an urge to check the phone). Accordingly, the majority of studies believe that the effect of the phone’s presence is inextricably linked to the fact that cell phones allow people to access social networks. Moreover, given that the familiarity of an object alters the amount and priority of attention that our cognitive system devotes to it in our visual environment (Wang et al., 1994; Jackson and Raymond, 2006), the effects of the phone’s presence may be derived from the specificity of cell phones (e.g., overexposure to cell phones that exist in the environment and long-term usage of cell phones with high frequency). This implies that these effects may be particular to the cell phone, which is regarded as the most common device used for instant communication; therefore, more researchers are aware that even cell phones that are not in use should be viewed as distractions in work and academic environments.

The cell phone is problematic because it is seen as an environmental stimulus that can trigger negative effects by its presence alone; however, this effect does not appear to be constant in varied situations and might be moderated by its activated state. The majority of studies have discussed the impact of the presence of the cell phone in various conditions in terms of visual saliency (e.g., the left side of the computer screen, the corner of the desk) and demonstrated that the presence of the visually prominent cell phone can significantly impair the efficiency of a visual search task (Ito and Kawahara, 2017) and cognitive tasks that assess general cognitive/attention functions (Thornton et al., 2014). In a similar situation, Ward, Duke, Gneezy, and Bos found that participants with their phone on the desk or in a bag/pocket performed the cognitive task (Unsworth et al., 2005) better than when the phone was taken away to another room (Ward et al., 2017). When the phone was taken into another room, the participants could not perceive whether it was activated compared to when it was close to them, which implies that the perception of the state of the cell phone probably moderated the intensity of the effect of the phone’s presence. On the other hand, it was demonstrated that the cell phone attracted individuals’ attention by ringing or notifying them about incoming information, which impaired their performance in a cognitive task (Stothart et al., 2015) and a driving task (Kass et al., 2016). In this case, the phone’s ringing and other alert sounds made it clear to the participants that the phone was in the room and emphasized its presence, implying that the enhanced awareness of the phone’s activeness caused the phone to interfere with the task at hand. Furthermore, compared to the participants who were banned from using cell phones, participants who were allowed to use cell phones performed worse on the multiple-choice test even in the case where they did not actually use their cell phones (Lee et al., 2017). This suggests that cell phone activeness is enhanced even without actual contact with the cell phone. These previous studies suggest that cell phones probably induce a cognitive cost related to task-irrelevant thought and that their activeness can be an important factor that changes the magnitude of the effect of cell phone presence.

Previous studies found evidence of the detrimental effect of the cell phone on working memory (Ward et al., 2017), visual-spatial search (Ito and Kawahara, 2017), and attention (Thornton et al., 2014), and this effect is thought to be linked to the reduction of available attentional resources caused by the presence of the cell phone (Ward et al., 2017). Meanwhile, several studies that reported a Null effect of the presence of a cell phone on the performance of other cognitive tasks while assessing different cognitive functions (e.g., inhibition, Johannes et al., 2018) and similar cognitive functions within different domains (e.g., short-term memory and perspective memory, Hartmann et al., 2020). The discrepancy in the results may be due to the extent to which the cell phone attracts attention; therefore, it is necessary to examine the effect of phone presence by systematically manipulating the degree to which the cell phone attracts attention.

### This Research

The aim of this study was to investigate whether the cell phone activeness manipulated by the state of the cell phone and the state of contact with the phone could moderate the effect of the phone’s presence by using the dual-task paradigm. This paradigm is based on the previous study (Liu et al., 2021) which assured the effect of the presence of a cell phone can be observed in the secondary task performance. In this paradigm, participants with a fixed gaze point conduct two tasks with different demands on attentional resources simultaneously (see Section “Experimental Task”). In order to complete the primary task (letter recognition task), the attentional resources need to be focused on the central visual field with a relatively low cognitive demand. While the secondary task (luminance detection task) was used to assess the extent of attentional resources spread around the peripheral visual field. That is, lower performance in a location implies that the amount of attentional resources allocated to that location has declined. A general characteristic of the attentional resources’ distribution in the peripheral visual field is that the further away attentional resources are from the central visual field, the less they are allocated (Miura, 1996). Due to this characteristic, the influence of reduced attentional resources distributed in the peripheral visual field is more likely to be found at locations far from the central visual field. It was plausible that the effect of the presence of a cell phone can be different across the eccentricity.

While previous studies have suggested that phone activeness is an important factor that changes the magnitude of the cognitive cost induced by cell phone presence, they do not have a uniform definition for cell phone activeness. Consequently, even though the manipulations that provide external stimuli on the phone such as notifications (Stothart et al., 2015) and adjusting the power state of the phone (Ward et al., 2017; Canale et al., 2019), aim to change the phone activeness, their effects are usually discreetly considered as two qualitative ones, based on the difference in the presence or absence of additional external stimuli (Ward et al., 2017). However, these types of manipulations should be counted according to the degree of potential for the phone to offer information to the participant if the activeness is indeed important. Therefore, in this study, phone activeness was defined as the subjective awareness of the phone’s activity and varied according to how much information could be offered *via* the experimental manipulations (e.g., a powered-off phone, powered-on phone, and powered-on phone with notifications can be regarded as not active, low active, and high active, respectively).

The power state of the phone is one of the potentially influential factors that can affect the phone activeness. To our knowledge, two studies discussed this factor but reported inconsistent findings. Canale et al. (2019) found that participants with their phones on silent mode performed the visual working memory task worse than those with a powered-off phone; however, Ward et al. found that the silent mode phone did not moderate the detrimental effect of the phone’s presence on the performance of cognitive tasks that assess working memory capacity (Baddeley et al., 2020), even in the high salience condition (i.e., placed on the desk, Ward et al., 2017). It is thus possible that the power state can moderate the phone activeness but not sufficiently enough to let the phone’s detrimental effect manifest itself at the behavioral level. Indeed, it cannot be ruled out that if participants know in advance that no messages will be sent to their phones, the phone may become less attractive. To complement this insufficient point, the phone activeness can be strengthened by a signal, such as a call or notification, that may increase the effect of the phone’s presence. This approach was proven to be effective in the study which found that the presence of a cell phone in silent mode with a brief notification (compare to the powered-off phone condition) impaired the performance of the SART task that assesses sustained attention (Stothart et al., 2015). Based on these investigations, we hypothesized that the effect of the phone’s presence would be increased by manipulating the phone activeness by the power state of the phone (H1).

In fact, people’s awareness of cell phones as active is reinforced by haptic stimuli as well as visual and auditory stimuli. Typically, to avoid missing notifications, people hold their phone without looking at it or put it in their pocket. In these situations, the notification in silent mode (powered-on) is usually accompanied by a vibration that allows incoming messages to be noticed even when the phone is not visible. Indeed, a haptic stimulus can attract attention as well as a visual stimulus (Spence and MaGlone, 2001) which can be why you can know when a message is coming in while holding the phone in your hand. Importantly, it is harder for people to shift their attention away from the tactile modality once it is focused there than it is to move the focus of their attention away from the auditory or visual modalities (Spence et al., 2001a,b). Therefore, we hypothesized that perceiving the presence of a cell phone through touch and receiving a haptic notification would draw more attention and lead to greater cognitive cost (H2). Therefore, this study tests this theoretical possibility to further understand the effects of the presence of a cell phone.

## Experiment 1

### Materials and Methods

#### Participants

A total of 25 students (males = 10, females = 15; *M*_*age*_ = 22.44, *SD*_*age*_ = 2.88) participated. All participants reported that their vision was at least 20/40. This study was approved by the Behavioral Research Ethics Committee of the School of Human Sciences at Osaka University in Japan (HB30-056). Informed consent was obtained from all participants after they read an information sheet that briefed them on the study.

We conducted a power analysis using G*Power 3.1.9.4 (Faul et al., 2007). A sample size of *N* = 20 was required to detect an effect size of Cohen’s *f* = 0.25 (partial η^2^ = 0.059) for the two-way interaction between power state and contact state on reaction time at a significance level of α = 0.05 and power of 1 - β = 0.80. We thus collected data from 25 individuals to prevent unavoidable data loss.

#### Experimental Task

A dual-task paradigm was employed. The primary task (letter recognition) was presented in the central visual field, and the secondary task (luminance change detection) was presented in the peripheral field of view (see the detail of stimulus arrangement in the Section “Apparatus and Stimuli”). In the dual-task paradigm, the stimuli of both tasks were presented simultaneously, but their corresponding responses needed to occur in sequential order (i.e., respond to the primary task first and then respond to the LCD task).

##### Letter Recognition Task

In the letter recognition task (also called the 0-back task, as a working memory measure; Kane and Engle, 2002; Conway et al., 2005), letters were presented sequentially, and participants needed to identify whether the current letter was not “A.” Participants were required to respond by pressing the “J” or “L” key with their right index and ring fingers if the letter was the same or different, respectively.

##### Luminance Change Detection Task

In the LCD task, the luminance change (from 24 to 11.5 cd/m^2^) occurred randomly in half of all the trials as selected by the system from the 12 dots located radially from the central point (see Section “Apparatus and Stimuli”). The participants were required to use their right thumb to press the “space” key if a change in luminance occurred. If a change did not occur, it was not necessary to press any key to signal a response.

#### Apparatus and Stimuli

The screen (IZUMI, AP − 100; 1987 [W] × 1490 mm [H]) used to present the stimuli was located 200 cm away from the participant’s seat. Stimuli were presented in the center of a screen (46 cm × 46 cm). A designated cell phone prepared by the experimenter (Apple iPhone 6s) was hung on the table in front of the participant’s seat using a flexible tablet arm (MAYOGA, B0739WLRD4) so that the device adjoined the left edge of the presented stimuli at a location with a visual angle of almost 9° when observed by the participants. Stimuli were presented on the screen and controlled by a computer (Dell, Vostro 5568) that operated the software PsychoPy (ver. 1.83.04). Responses were collected *via* a keyboard (DELL SK-8115) connected to the computer.

**FIGURE 1:**
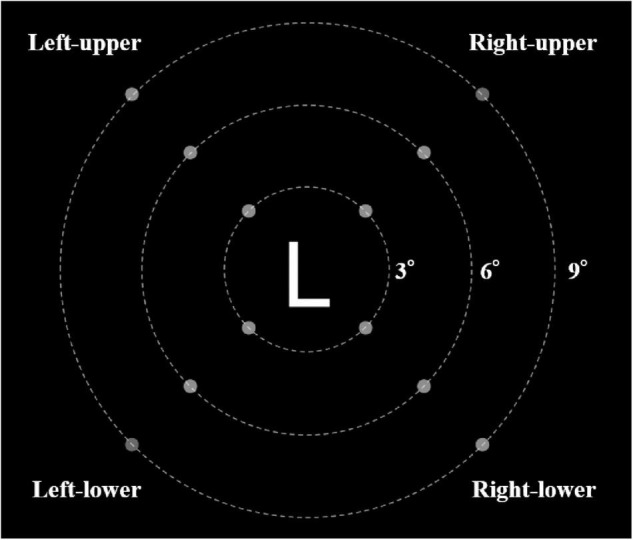
The layout of stimulus (The white dashed line in the figure does not appear in the experimental task).

The stimulus layout is shown in Figure 1. The stimulus of the letter recognition task was presented in the central visual field, and the stimulus of the luminance change detection task was presented in the peripheral field of view. A letter (2.2° × 2.2°, 88 cd/m^2^) was presented in the center of the screen, and a total of 12 dots were presented in the peripheral field of view. The dots were 0.55° in diameter at a visual angle and allocated on three concentric circles (eccentricity: 3°, 6°, and 9° in diameter). Each of the four dots in the circle was 90° apart and 45° from the horizontal-vertical direction.

#### Procedure

After completing the informed consent form, the participants sat with their heads in a fixed chin-rest. Their eyes look horizontally right at the fixation point (+). Before initiating the primary task session, participants attended a practice session in order to ensure that they understood the correct way to respond and had no vision problems that could impair their ability to detect the luminance change.

**FIGURE 2:**
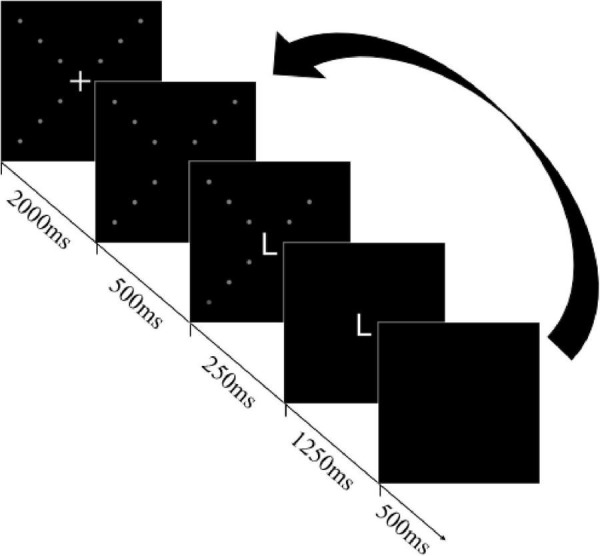
A sequence in a single trial (luminance change in this example occurred in the right-upper quadrant at an eccentricity of 9°).

In the primary task session, after participants pressed the space key when they were ready to begin, the fixation point (+) and dots were presented. After 2000 ms, the fixation point disappeared, and the initial trial began. The procedure for single trials is shown in Figure 2. The dots were continuously presented for 500 ms, and then the letter was presented in the center. In half of the trials, the luminance change of a dot would occur while the letter was presented (the duration of the change in luminance was 250 ms). In the other half of the trials, no luminance change occurred during the 250 ms duration. Whether the luminance change occurred or not, all of the dots disappeared after 250 ms from the letter was presented. In the meantime, the letter was continuously presented for 1,250 ms before disappearing (the letter’s total presentation duration was 1,500 ms). Participants were asked to respond to both tasks before the next trial began. Regardless of whether participants responded to both tasks or not, the next trial began when the time was up.

All participants took part in a total of six experimental conditions (see Section “Experiment Design”) and were given a 4 min rest at the end of every condition. The order of the trials in every condition was randomized. To be more specific, three experimental conditions were conducted in a contact condition (i.e., the phone is held in the left hand), and the others took place in a no-contact condition (i.e., the phone is placed on the left side of the keyboard). In the no-contact condition, the location of the participant’s left hand was not specified, while in the contact condition, the participants were instructed to place their left hand holding a cell phone in the location where the phone was placed in the no-contact condition. Both sets of three conditions were completed in order of cell phone state condition (mobile-battery, powered-off, powered-on), respectively. A mobile-battery condition was a control condition designed to reveal how participants conducted the task in a situation without a cell phone. In the mobile-battery condition, a mobile battery that was almost the same size and weight as the cell phone was used to make the physical situation the same as the other two phone conditions. In a powered-off condition, the real phone was placed in the same location, but the power was turned off. In a powered-on condition, the real phone was placed in the same location, but its power was turned on. In addition, the phone notifications (visual stimulus) occurred three times during a single block task. Participants were told that the phone in a powered-on condition was enabled to receive notifications, but if any notification came in, it was not related to the experimental task. Participants were debriefed at the end of the experiment.

#### Experiment Design

This experiment was a 2 (the state of contact: no-contact or contact) × 3 (dots distance: 3, 6, 9) × 3 (the state of phone: control, powered-off, powered-on) factorial design. In each experimental condition, the luminance change occurrence rate at each light spot position was 50%. In total, luminance changes occurred in 864 trials, and in the other half of the trials, the luminance change did not occur. In total, the experimental sessions consisted of 1,728 trials (144 trials per block) preceded by 194 practice trials.

### Results

#### Data Analysis

One participant pressed the wrong key in one block, so the data of this participant were discarded, and the remaining 24 participants’ data were analyzed. Participants were instructed to respond to the primary task first, so the data of trials in which participants first responded to the secondary task were discarded (176 trials). The trials that took place during the notifications in the powered-on condition were discarded. The remaining trials (97.84% of all trials) were used for the analysis.

The angular transformation of accuracy (the ratio of hit and correct rejection) and the reaction time of the letter recognition task were analyzed using a two-way ANOVA with the state of contact and the state of the phone as within-participant factors. The angular transformation of the hit rate of the LCD task was analyzed using a three-way ANOVA with dots distance, the state of contact, and the state of the phone as the within-participant factors. All degrees of freedom were adjusted by using Chi-Muller’s epsilon. All the multiple comparisons used Shaffer’s multiple-comparison procedure.

#### Letter Recognition Task

**FIGURE 3:**
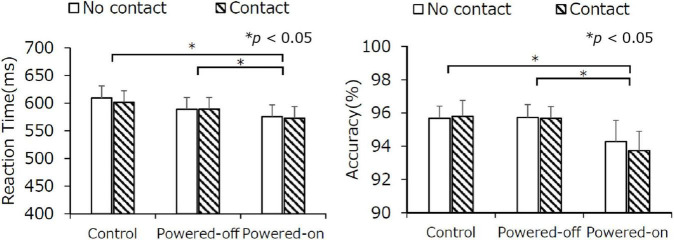
The reaction time (left) and accuracy (right) of the letter recognition task in experiment 1. Error bars depict standard error values.

Reaction time results are shown in Figure 3 (left). The main effect of the state of the phone on reaction time was significant, *F*_(1.26,29.07)_ = 6.20, *p* = 0.01, η^2^ = 0.0154, but there was no other significant main effect or interaction. The reaction time was faster for the powered-on condition (574 ms) than it was for the powered-off condition (589 ms) and the control condition (606 ms; *ps* < 0.05), which revealed that participants responded faster when they were in the presence of a powered-on phone. The main effect of the state of the phone on accuracy was significant, *F*_(2,46)_ = 4.33, *p* = 0.02, η^2^ = 0.0243. The paired comparisons indicate that the accuracy in the powered-on condition was lower than it was in the control and powered-off conditions (*ps* < 0.05). No other significant main effect or interaction in accuracy was detected (Figure 3, right).

#### Luminance Change Detection Task

**FIGURE 4:**
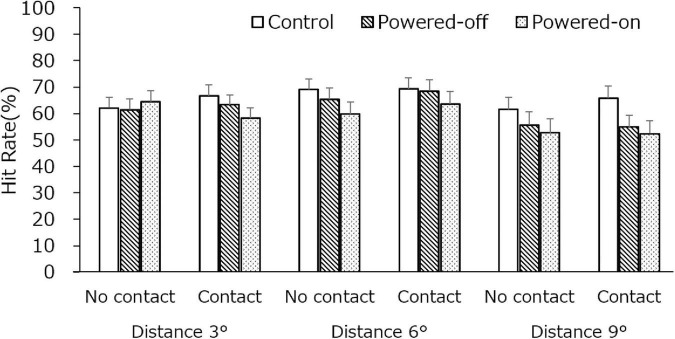
The hit rate of LCD task in experiment 1. Error bars depict standard error values.

The results are depicted in Figure 4, and detailed data are shown in Table 1. The main effects of the state of the phone, *F*_(1.51,36.22)_ = 11.03, *p* < 0.001, η^2^ = 0.0190, and dots distance, *F*_(1.17,28.01)_ = 7.96, *p* < 0.01, η^2^ = 0.0281, were significant. The two-way interaction with the state of the phone and dots distance was significant, *F*_(4,96)_ = 6.80, *p* < 0.001, η^2^ = 0.0052. The three-way interaction was also significant, *F*_(4,96)_ = 5.80, *p* < 0.001, η^2^ = 0.0040. No other significant main effect or two-way interaction was observed.

**TABLE 1:** The hit rate detail across the experimental conditions in experiment 1.

	Control		Powered-off		Powered-on	
			
	Contact	No contact	Contact	No contact	Contact	No contact
Dots distance 3	66.67%	62.14%	63.37%	61.46%	58.23%	64.48%
Dots distance 6	69.27%	69.09%	68.58%	65.35%	63.53%	59.79%
Dots distance 9	65.80%	61.55%	54.86%	55.73%	52.40%	52.71%

We tested for a simple interaction between the state of the phone and the state of contact at each level of factor dots distance. At dots distance 3, the simple main effects of the state of the phone and the state of contact were not significant (*p*s > 0.10), but the simple interaction of the state of the phone and the state of contact was significant, *F*_(1.83,43.82)_ = 5.05, *p* = 0.01, η^2^ = 0.013. A significant difference between the state of the phone was only observed in the contact condition in which the hit rate in the control condition (66.7%) was higher than it was in the powered-on condition (58.2%; *p* < 0.05). In addition, when the powered-on phone was in a no-contact condition, the hit rate (64.5%) was higher than it was in a contact condition (58.2%; *p* < 0.01). At dots distance 6, only the simple main effect of the state of phone was significant, *F*_(1.32,31.75)_ = 11.60, *p* < 0.001, η^2^ = 0.023. The hit rate in the control (69.1%) and powered-off (67.0%) conditions was higher than it was in the powered-on condition (61.7%; *p*s < 0.05). At dots distance 9, only the simple main effect of the state of the phone was significant, *F*_(1.83,43.84)_ = 16.04, *p* < 0.001, η^2^ = 0.041. The hit rate in the control condition (63.7%) was higher than it was in the powered-off (55.3%) and powered-on (52.6%) conditions (*p*s < 0.05).

We tested for a simple interaction between the state of the phone and dots distance at each condition of the state of contact. In the no-contact condition, the simple main effect of the state of the phone was marginally significant, *F*_(1.49,35.87)_ = 3.71, *p* = 0.05, η^2^ = 0.011, and the dots distance was significant, *F*_(1.22,29.31)_ = 5.39, *p* = 0.02, η^2^ = 0.024. The simple interaction of the state of the phone and dots distance was also significant, *F*_(4,96)_ = 8.12, *p* < 0.001, η*^2^* = 0.012. The state of the phone was different in dots distance 6, where the hit rate in the control condition (69.1%) was higher than in the powered-off condition (65.4%) which was higher than in the powered-on condition (59.8%; *p*s < 0.05). The hit rate in the control condition (52.78%) was higher than it was in the powered-off (49.03%) and powered-on conditions (46.82%) in dots distance 9 (*p*s < 0.05). The changes between the different dot distances showed that the hit rate at distance 6 was higher than it was at distances 3 and 9 (*p*s < 0.05) in the control condition. The hit rate at distance 6 was higher than it was at distance 9 (*p* < 0.05) in the powered-off condition, and the hit rate at distances 3 and 6 was higher than it was at distance 9 (*p*s < 0.05) in the powered-on condition. In the contact condition, the simple main effect of the state of the phone, *F*_(1.64,39.44)_ = 8.76, *p* < 0.01, η^2^ = 0.029, and the dots distance was significant, *F*_(1.21,29.02)_ = 10.49, *p* < 0.001, η^2^ = 0.032. The simple interaction of the state of the phone and dots distance also was significant, *F*_(3.7,88.82)_ = 4.56, *p* < 0.01, η^2^ = 0.007. At distance 3, the hit rate in the control condition (66.7%) was higher than it was in the powered-on condition (58.2%; *p* < 0.05). At distance 9, the hit rate in the control condition (65.8%) was higher than it was in the powered-off (54.9%) and powered-on (52.4%) conditions (*p*s < 0.05). The changes between the different dot distances showed that the hit rate at distances 3 and 6 was higher than it was at distance 9 (*p*s < 0.05) in the powered-off condition, and the hit rate at distance 6 was higher than it was at distance 9 (*p*s < 0.05) in the powered-on condition.

We tested for a simple interaction of the state of contact and dots distance at each condition of the state of the phone. In the control condition, the simple main effect of dots distance was significant, *F*_(1.26,30.35)_ = 4.26, *p* = 0.04, η^2^ = 0.015. The hit rate at distance 6 was higher than it was at distances 3 and 9 (*p*s < 0.05). In the powered-off condition, the simple main effect of dots distance was significant, *F*_(1.39,33.32)_ = 10.57, *p* < 0.01, η^2^ = 0.053. The hit rate at distance 6 was higher than it was at distances 3 and 9 (*p*s < 0.05). In the powered-on condition, the simple main effect of dots distance was significant, *F*_(1.21,29.06)_ = 7.38, *p* < 0.01, η^2^ = 0.035, and the simple interaction of the state of contact and dots distance was significant, *F*_(1.92,46.12)_ = 9.74, *p* < 0.001, η^2^ = 0.010. The hit rate at distances 3 and 6 was higher than it was at distance 9 (*p*s < 0.05) when the phone was placed on the side, while the hit rate at distance 6 was higher than it was at distances 3 and 9 (*p*s < 0.05). The hit rate in the no-contact condition was higher than it was in the contact condition at distance 3, but the opposite result (the hit rate in the contact condition was higher than it was in the no-contact condition) was observed at distance 6 (*p*s < 0.05).

### Discussion

The results of experiment 1 indicated that the presence of a cell phone impaired the participants’ performance on LCD task that is sensitive to available attentional resources. Moreover, the decreased hit rate of LCD task in powered-off condition (vs. mobile battery condition) showed at dots distances 6, 9 than dots distance 3, indicating that the reduction in attentional resources caused by the presence of the phone was more pronounced at locations far from the central visual field. The influence of the phone state (i.e., the difference between powered-on and powered-off conditions) was found in the situation where the phone was placed to the side while the luminance changes occurred at dots distance 6. This result indicated that the phone state can enhance the awareness of the phone activeness, rendering greater deterioration in the hit rate of detecting luminance changes. Regarding the influence of physical contact with the phone, it was found that the participants who had the phone to the side (vs. holding it in their hand) showed better detection performance when the luminance changes occurred at dots distance 3, but worse detection performance when the luminance changes occurred at dots distance 6. These results revealed that physical contact with the phone might moderate the effect of the presence of a cell phone, but its directionality is unclear. During the letter recognition task, the participants responded faster, but their accuracy lessened in the powered-on conditions (vs. the powered-off and control conditions), which indicates that the presence of the cell phone might affect reaction strategies but not the total amount of attention directed toward the entire task.

These findings supported our assumption that phone activeness can moderate the amount of attentional resources occupied by the presence of a cell phone. However, the influence of the state of the phone was significant only in the limited condition (i.e., no contact and dots distance 6 condition) while the influence of contact with the phone was not significant. As shown in the descriptive data, the hit rates in the LCD task across the dots distance conditions as well as the state of contact conditions in the powered-off condition were higher than in the powered-on condition (except for the no-contact and dots distance 3 condition). The reason for the somewhat inconsistent effect of the phone’s state may be caused by a large individual difference in the ability to detect luminance changes. Indeed, the hit rates in the LCD task ranged widely, indicating that the task was very difficult for some participants and very easy for others. Due to the wide variation in task difficulty among participants, it might be possible that the difference between the state of the phone conditions became difficult to be detected. Therefore, the results of experiment 1 are not sufficient enough to examine our hypothesis that the effect of the presence of the phone is affected by the cell phone activeness, and it is necessary to examine whether these results can be replicated when the task difficulty is adjusted to be constant for all participants.

## Experiment 2

The results of experiment 1 support the proposition that the presence of a cell phone reduces available attentional resources, and its detrimental effect was affected by the power state and the contact state of the phone. However, the effects of the phone’s presence appear to be limited by the distance from the center at the stimulus location, and the moderated effects caused by the state of the phone and contact with the phone were indiscernible. This ambiguity may stem from individual differences in the ability to detect luminance changes. In experiment 1, some participants might have found it too easy or too difficult to detect the luminance change in some dot positions, which means that task performance might have not been measured appropriately enough. Therefore, in experiment 2, we replicated the basic design of experiment 1 and ruled out the potential influence of this individual difference in sensitivity to luminance changes by adjusting the dot’s luminance so that all participants can detect the luminance change appropriately.

### Materials and Methods

#### Participants

A total of 25 students (males = 16, females = 9; *M*_*age*_ = 23.06, *SD*_*age*_ = 1.62) participated. A total of 24 of the participants reported normal or corrected-to-normal vision (at least 30/40). The one participant who did not have 30/40 vision performed the practice trial as well as the other participants. This study was approved by the Behavioral Research Ethics Committee of the School of Human Sciences at Osaka University in Japan (HB019-007). Informed consent was obtained from all participants after they read an information sheet that briefed them on the study. The sample size was the same as it was in experiment 1.

#### Threshold Task and the Performance Confirmation Task

The purpose of the threshold task was to obtain the parameter value of the luminance change amount when the hit rate at dots distance 9 was equal to the chance level (50%) by measuring the hit rate of luminance changes with different luminance change amounts. The changing luminance range was between (0.3 cd/m^2^) and (78.93 cd/m^2^), and the amount of luminance change in this range was divided into 10 equal portions. The ten conditions with different luminance change amounts were repeated ten times. The procedure of the threshold task was identical to the main experimental task, except that the luminance changes only appeared in four locations at dots distance 9. Moreover, 80 trials (20% of threshold test trials) without luminance changes were also added as dummy trials in order to prevent the participants from being aware that luminance changes should occur in every trial (total trials: 480).

We fit a hit rate curve range from hard to easy to each individual, based on their result of threshold task (criterion was adjusted to 50% depending on change level) by using the Generalized Linear Model (GLM, the fixed factors: the portion; the distribution of dependent variable: binomial; the link function: logit). The luminance change amount corresponding to the hit rate of 50% at dots distance 9 was calculated. The reliability of the obtained parameter value was retested in the performance confirmation task. This manipulation ensured that the sensibility of the luminance change at 9° was systematically equal among all the participants.

#### Procedure

All the participants took part in a 2-day experiment. The threshold task was conducted on the first day, and the main experiment task took place on the next day (the procedure of the main experiment task was identical to the one used for experiment 1).

In threshold task, the participants performed three sessions (the practice session, the threshold task, and the performance confirmation task). Participants attended the practice session (2 blocks, 144 trials per block). These sessions were provided to ensure that the participants already knew which responses were correct and that they did not have any visual problems that could impair their ability to detect the luminance change. Then they performed the threshold task (2 blocks, 240 trials per block), and the hit rate at the eccentricity of 9° was calculated. The obtained parameter values were used in the performance confirmation task (144 trials; identical to one block in experiment 1) to confirm whether the threshold calculation was accurate as expected. The threshold task and the performance confirmation task followed the identical procedure used in the main experiment task, except with respect to the amount of change in luminance. This part of the experiment took approximately 90 min.

### Results

#### Data Analysis

The data from one participant could not be collected due to a program error. So, the data of this participant were discarded, and the remaining 24 participants’ data were analyzed. The data of the trials were discarded using the same criteria that were adopted in experiment 1. Then, the remaining trials (98.77% of all trials) were used for analysis. The procedure used to analyze the data of the main tasks was the same as the one used in experiment 1.

#### The Performance Confirmation Task

**FIGURE 5:**
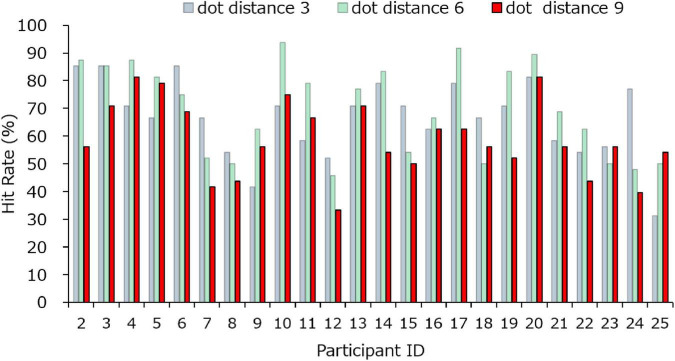
The hit rate of confirmation task per participants in experiment 2.

The result of the performance confirmation task is shown in Figure 5. It indicated that the range of hit rate was 33.3–81.3%. A total of 19 participants achieved a hit rate of more than 50% at the eccentricity of 9°, and the 5 participants had a hit rate of less than 50%. The average hit rate of all participants at 3, 6, and 9 was 66.49, 69.79, and 58.85%, respectively. The standard deviation of the hit rate at dots distance 9 in experiment 1 ranged from 22.40% (phone control condition in no contact condition) to 25.91% (powered-on phone condition in no contact condition). While the standard deviation in the performance confirmation task was 13.4%. The threshold adjustment decreased the difference in the ability to detect luminance changes between participants, therefore, it can be assumed that the obtained parameter of luminance change amount is appropriate for the next main experiment task.

#### Letter Recognition Task

**FIGURE 6:**
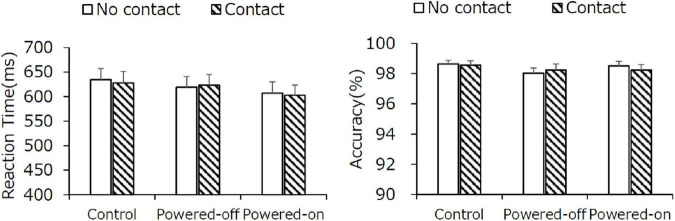
The reaction time (left) and accuracy (right) of the letter recognition task in experiment 2. Error bars depict standard error values.

The results of the letter recognition task are shown in Figure 6. The main effect of the state of the phone was significant in reaction time, *F*_(1.79,41.25)_ = 4.25, *p* = 0.02, η^2^ = 0.010, but paired comparisons indicated that the difference in reaction time between the state of the phone conditions was not significant. No other significant main effect or interaction in reaction time and accuracy was observed. The speed-accuracy trade-off in the powered-on condition that was observed in experiment 1 did not occur in experiment 2.

#### Luminance Change Detection Task

**FIGURE 7:**
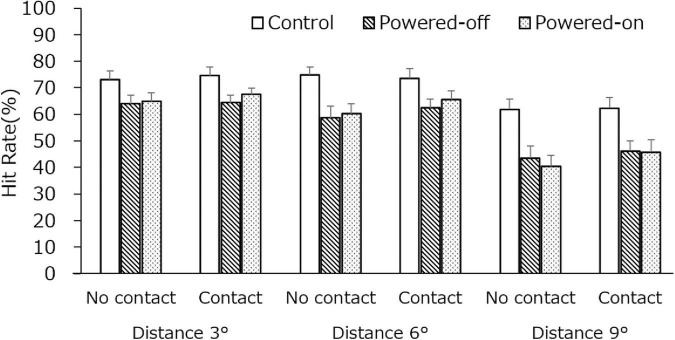
The hit rate of the LCD task in experiment 2. Error bars depict standard error values.

The results of the LCD task are shown in Figure 7. The main effects of the state of the phone, *F*_(2,46)_ = 19.90, *p* < 0.001, η^2^ = 0.096, and dots distance, *F*_(1.36,31.29)_ = 43.00, *p* < 0.001, η^2^ = 0.149, were significant. The two-way interaction with the state of the phone and dots distance was significant, *F*_(3.57,82.16)_ = 4.34, *p* < 0.01, η^2^ = 0.008. At all dots distances, the hit rate in the control condition was higher than it was in the powered-off condition and powered-on conditions (*p*s < 0.05). Regarding the difference in dots distance in all of the state of phone conditions, the hit rate at distances 3 and 6 was higher than it was at distance 9 (*p*s < 0.05). No significant difference between the state of contact and the state of the phone was observed, which was unexpected. Nevertheless, the descriptive statistics (see Table 1) indicate that the hit rates in almost all of the experimental conditions were better when the participants had contact with the phone than when they did not.

### Discussion

The result of experiment 2 replicated the finding that the presence of a cell phone impaired the performance on the LCD task. The detrimental effect of the phone’s presence was observed more obviously than it was in experiment 1 in that the hit rate for all dots distances was lower when the phone was present compared to when the mobile battery was presented. This bears out our speculation that individual differences in the ability to detect luminance changes do affect the significance of the cell phone presence effect on LCD task performance.

However, contrary to the findings of the influence of the power state and phone contact that emerged in experiment 1, the results of statistical analysis showed the null effects of these factors were observed in experiment 2. Despite that, the descriptive statistics (Table 2) showed that the hit rates that were recorded when participants made contact with the phone under all conditions were higher than the hit rate that was recorded when participants were not in contact with the phone. This tendency was not observed significantly in statistics, nevertheless, it helped to clarify the directionality of the moderated effect of making contact with a cell phone. It was possible that the lack of statistical significance of the state of contact condition may be due to the multi-factors involved in the analysis. Indeed, the results revealed that the hit rate of LCD task was influenced by the presence of a cell phone itself much more strongly, rather than by the state of contact. It indicated that the influence of contact with the phone, if any, may be undetectably weak among other stronger effects. Moreover, there was no consistent tendency about the power state of the phone condition in both statistical and descriptive results, so it should be treated as a null effect. It indicated that the LCD task results about the state of the phone in experiments 1 and 2 were not consistent. Hence, the difference in hit rate between the powered-off and powered-on conditions which were observed in experiment 1 should be treated carefully, and further scrutiny is needed.

**TABLE 2:** The hit rate detail across the experimental conditions in experiment 2.

	Control		Powered-off		Powered-on	
			
	Contact	No contact	Contact	No contact	Contact	No contact
Dots distance 3	74.59%	72.99%	64.50%	63.93%	67.58%	64.83%
Dots distance 6	73.43%	74.84%	62.38%	58.74%	65.50%	60.18%
Dots distance 9	62.31%	61.93%	46.19%	43.54%	45.78%	40.36%

## General Discussion

This study investigated whether the power state of the phone and physical contact with the phone moderate the effect of the phone’s presence. Through two experiments, we found that the participants in the situation where the phone was present performed the LCD task worse than the participants with a mobile battery nearby, which indicates that the presence of a cell phone can decrease available attentional resources. However, the decrement in the LCD task performance was not consistently changed either by the change in the power state or by the change in the physical contact state. Although some evidence from experiment 1 showed that the state of the phone and the state of contact manifested effects, these results were not observed in experiment 2. Therefore, based on the current findings, we cannot clearly speak in favor or against our prediction that the negative effect of the presence of a cell phone is reinforced by the state of the phone (H1) or the state of contact (H2).

Consistent with the prior studies (Thornton et al., 2014; Ito and Kawahara, 2017; Ward et al., 2017; Canale et al., 2019; Tanil and Yong, 2020), we found a negative effect of the presence of a cell phone on the overall performance of the LCD task. While a simple detection task that involves perceiving luminance changes and responding by pressing a key is based on simple perceptual and motor processes, more complex cognitive processes are needed to perform the letter recognition task, such as recognizing what letter is presented and judging whether it is A or not. The efficiency of the detection depends on whether there is a minimum amount of attentional resources distributed at the location where the change occurs. Since minimal attentional resource allocation is required to perform change detection, poor change detection performance indicates that few attentional resources are being directed to the location of the change. Therefore, it means that allocation of spatial attention has been impaired by the presence of a cell phone.

Meanwhile, contrary to the significant difference that emerged in LCD task performance, the cell phone’s influence on the letter recognition task performance was vague. In the power-on condition in experiment 1, the reaction time was shorter, but the accuracy was lower, reflecting a speed-accuracy tradeoff (Wickelgren, 1977). The speed-accuracy tradeoff is often considered to be an alteration in strategy independent of the attention allocation policy; hence, the overall amount of attentional resources can be deployed in conducting the letter recognition task was similar across the state of phone conditions. In experiment 2, on the other hand, there was a weak tendency toward shorter reaction times as the phone activeness was increased. These results showed a rather different directionality of performance change in the letter recognition task that appears to be more of a beneficial effect than we would expect (it should be noted that the result was not statistically significant). Since a similar directionality has been shown in the previous study in which almost the same task was used (Liu et al., 2021), this positive effect might be regarded as a somewhat robust phenomenon. Combined with the corresponding response to the LCD task, the current findings might reveal a weak promotional effect on the reaction time for the letter recognition task response accompanied by a significant decrease in the detection efficiency of peripheral luminance changes. In the case of dual-task paradigm, the stimuli of both tasks were presented simultaneously, the attentional resources devote to the perceptual processing of these stimuli can be mutually constrained (more allocation on one side, less allocation on the other). That is, even if the available attentional resources are reduced in the dual-task paradigm situation, the performance of at least the prioritized task does not necessarily decrease due to the adjustment of participants’ attentional resource allocation (Norman and Bobrow, 1975). Hence, it may be that the presence of a cell phone affected the performance in both tasks, but only the secondary one manifested a negative effect due to the dynamic attention allocation moderated by the participants’ strategy. Given that the participants were required to preferentially respond to the letter recognition task, they may have unconsciously focused more attentional resources on the center area where the primary task stimulus was presented to mitigate the negative effect induced by the presence of a cell phone, rendering the low performance on the secondary LCD task more pronounced. If this is the case, we could not determine whether the presence of a cell phone also has a negative impact on the letter recognition task based on the current findings alone due to the fact that the letter recognition task and LCD tasks are not completely independent. Further investigation is needed to examine how the presence of a cell phone manifests an effect when the two simultaneous tasks have equal priority.

Another crucial question that we investigated in this study was whether the negative effects of the presence of a cell phone are more serious when the phone activeness is enhanced. With respect to the effects of the state of the phone, we found that the state of the phone decreased the performance in detecting a luminance change at dots distance 6 when they were not in contact with the phone in experiment 1. While this decrement effect was not observed when the individual differences in the ability to detect luminance changes were controlled in experiment 2. The inconsistency of the results of experiment 1 and experiment 2 may be because the cell phone does not belong to the participants, hence the impact of cell phone power becomes vulnerable and more susceptible to other factors. Indeed, the existing literature highlights the link between the state of the phone (e.g., the phone salience, the availability of the phone) and its detrimental effect (Stothart et al., 2015; Ward et al., 2017; Canale et al., 2019). This suggests that the presence of one’s own cell phone in a powered-on state creates a greater cognitive cost, which was believed to be caused by phone-related thoughts that can emerge in the situation where a participant’s phone can receive messages. However, because the cell phones used in this study were not owned by the participants themselves, they may have had difficulty perceiving the notifications from their cell phones as being relevant to them. In order to verify the effect of the presence of a cell phone in real-life situations, it is necessary to use the participants’ own cell phones so that they will perceive the notifications as being personally relevant.

Regarding the impacts of contact with the phone, the results of experiment 1 showed that the slight effect of contact with the cell phone was observed but it was unclear whether it facilitated or interfered with detection task performance. While we attempted to figure out the directionality of the effect of contact with the phone in experiment 2, a tendency to facilitate luminance detection was observed (note that it was not statistically significant). This study was concerned about the impact of the presence of a cell phone in this physical contact situation (i.e., people are holding the phone when conducting a task) because it is very common in our lives. Our assumption was based on the theoretical thought regarding the impact on attentional capture by the haptic stimuli as well as the general belief that holding the phone in one’s hand is distracting. However, at least with respect to visual attention, the negative effects of being in contact with a cell phone may, if any, not be so large, implying that physical contact would not have as a serious negative impact, as the public believes.

Cell phone ownership varies across studies: In most cases, the phone belongs to the participant (experiment 2 in Thornton et al., 2014; Stothart et al., 2015; Ward et al., 2017; Johannes et al., 2018; Canale et al., 2019; Hartmann et al., 2020; Tanil and Yong, 2020), but there are some cases where it did not (Przybylski and Weinstein, 2013; experiment 1 in Thornton et al., 2014; Allred and Crowley, 2017; Ito and Kawahara, 2017; Crowley et al., 2018). In studies that examined the influence of the power state of a cell phone (Ward et al., 2017; Canale et al., 2019), the used cell phone was the participant’s own. Despite the fact that cellphone ownership might play a crucial role in these scenarios, the influence of this factor has not been considered enough in the literature to date. As mentioned above, using a cell phone without ownership might not be sufficient to induce the cognitive cost associated with task-unrelated thought. Consequently, it does not necessarily make the effect of the phone’s presence stronger, even though notifications further increase the phone activeness. In the current case, considering the use of cell phones in the real world, we should originally have used cell phones owned by the experimental participants themselves. However, the participants in the experiment had a wide variety of cell phones, and it was necessary to use a common cell phone as the experimental device in order to control the physical conditions. This is one of the important limitations of this study, as it may have greatly affected the results of this study, including the effect of the phone’s power state condition.

## Conclusion

In conclusion, this study demonstrated that the presence of a cell phone interfered with the deployment of attentional resources and decreased the efficiency of peripheral luminance change detection. This detrimental effect was not intensified by either the power state of the phone or whether the participant had physical contact with it; however, we did not reach any conclusions about whether cell phone activeness influences the negative impact of their presence. Nevertheless, on the issue of the interference from the presence of cell phones in the work environments, this study may suggest that even a minimal awareness of a phone’s presence (i.e., powered-off state) may cause a distraction, whether or not the device belongs to us or someone else. This implies that the presence of cell phones as information transfer devices in a highly informative work environment can become problematic.

## Data Availability Statement

The raw data supporting the conclusions of this article will be made available by the authors, without undue reservation.

## Ethics Statement

The studies involving human participants were reviewed and approved by Behavioral Research Ethics Committee of the Osaka University School of Human Sciences. The patients/participants provided their written informed consent to participate in this study.

## Author Contributions

WL contributed to the data acquisition and wrote the first draft of the manuscript. All authors contributed to the study conception and design, statistical analysis and discussing, and revising and reading the manuscript and approved the final version of the manuscript for submission.

## Conflict of Interest

The authors declare that the research was conducted in the absence of any commercial or financial relationships that could be construed as a potential conflict of interest.

## Publisher’s Note

All claims expressed in this article are solely those of the authors and do not necessarily represent those of their affiliated organizations, or those of the publisher, the editors and the reviewers. Any product that may be evaluated in this article, or claim that may be made by its manufacturer, is not guaranteed or endorsed by the publisher.
